# Integrative Metagenomics–Metabolomics for Analyzing the Relationship Between Microorganisms and Non-volatile Profiles of Traditional *Xiaoqu*

**DOI:** 10.3389/fmicb.2020.617030

**Published:** 2021-02-01

**Authors:** Chi Zhao, Wei Su, Yu Mu, Yingchun Mu, Li Jiang

**Affiliations:** ^1^School of Liquor and Food Engineering, Guizhou University, Guiyang, China; ^2^Guizhou Key Laboratory for Storage and Processing of Agricultural and Animal Products, Guizhou University, Guiyang, China

**Keywords:** traditional *Xiaoqu*, metabolomics, metagenomics, non-volatile metabolite, functional microorganism, metabolic pathway

## Abstract

*Xiaoqu*, one of three traditional *jiuqu* in China, is a saccharifying and fermenting agent used in *Xiaoqu jiu* brewing, with different ingredient compositions and preparation techniques used in various regions. The yield and quality of *Xiaoqu jiu* are significantly affected by the metabolites and microbiota of *Xiaoqu*; however, the associated relationship remains poorly understood. This study aimed to analyze this relationship in three typical traditional *Xiaoqu* from the Guizhou province in China. The non-volatile metabolites of *Xiaoqu* were detected using gas chromatography time-of-flight mass spectrometry, whereas the classification and metabolic potential of the microbiota were investigated using metagenomic sequencing. Results show that Firmicutes, Proteobacteria, and Actinobacteria represent the dominant bacterial phyla, with *Lactobacillus, Bacillus, Acinetobacter, Leuconostoc*, and *Weissella* found to be the dominant bacterial genera. Meanwhile, Ascomycota, Mucoromycota, and Basidiomycota are the dominant fungal phyla with *Aspergillus, Saccharomyces, Pichia, Rhizopus*, and *Phycomyces* being the predominant fungal genera. Functional annotation of the microbiota revealed a major association with metabolism of carbohydrates, cofactors, and vitamins, as well as amino acids. A total of 39 significantly different metabolites (SDMs) were identified that are involved in 47 metabolic pathways, primarily that of starch and sucrose; glycine, serine, and threonine; glyoxylate and dicarboxylate; pyruvate; as well as biosynthesis of pantothenate and CoA. Further, based on Spearman's correlation analysis, *Aspergillus, Saccharomyces, Lactobacillus, Acetobacter, Weissella, Pantoea, Desmospora*, and *Bacillus* are closely correlated with production of physicochemical indexes and SDMs. Moreover, the metabolic network generated for the breakdown of substrates and formation of SDMs in *Xiaoqu* was found to primarily center on the metabolism of carbohydrates and the tricarboxylic acid cycle. These results provide insights into the functional microorganisms and metabolic patterns present in traditional Guizhou *Xiaoqu* and might guide researchers in the production of stable and efficient *Xiaoqu* in the future.

## Introduction

Chinese *Xiaoqu jiu* accounts for approximately one sixth of the total liquor production in China and is widely distributed throughout the southern regions, including Guizhou, Sichuan, and Hubei provinces (Wang J. et al., 2018; Wang M. Y. et al., 2019). It is brewed using raw materials, such as rice, corn, wheat, sorghum, and *Xiaoqu*, which is a complex saccharifying and fermenting agent. Due to differences in ecological characteristics and technological parameters employed in the production of *Xiaoqu jiu*, various starters are employed in different regions, such as *Ragi* in Indonesia (Siebenhandl et al., 2001), *Men* in Vietnam (Dung et al., 2006), *Marcha* in India and Nepal (Sha et al., 2017), *Dombea* in Cambodia (Sokny et al., 2018), and *Nuruk* in Korea (Kim et al., 2019). *Xiaoqu*, specifically, is produced by solid-state fermentation comprising soaking, grinding, molding, powder coating, cultivating, ripening, and drying of rice and rice bran (Liu and Sun, 2018). During this procedure, numerous microbes, enzymes, aroma precursors, and significant aroma constituents are enriched, contributing to the style and quality of the final distillate (Gou et al., 2015).

Microorganisms are the key factors contributing to the delicate balance between stability, quality, and productivity of *Xiaoqu jiu*, making analysis of the core functional microorganisms the basis for realizing modern standardized *Xiaoqu* production (Wang X. D. et al., 2019). However, in most regions of Asia, including India (Bora et al., 2016), Thailand (Chuenchomrat et al., 2008), Korea (Son et al., 2018), and China (Zhao et al., 2020), *Xiaoqu* and *Xiaoqu jiu* are manufactured under conditions of spontaneous fermentation in an open environment based on operation skills and individual experiences. This often leads to inconsistent quality between batches and potential food safety concerns (Zheng and Han, 2016). The application of a standardized *Xiaoqu* protocol represents an effective way to regulate the fermentation process and regulate *Xiaoqu jiu* quality. Hence, investigation into the functional activity of microorganisms present in *Xiaoqu* is important.

With the development of detection technology, increasing focus has been placed on investigating the phenotypic correlation between microorganisms and metabolites with fermented products (Elhalis et al., 2020; Lv et al., 2020). Previous studies using amplicon sequencing technology identified *Weissella, Aspergillus, Rhizopus, Staphylococcus, Saccharomyces*, and *Candida* as the dominant microorganisms in traditional *Xiaoqu* starters in Sichuan, Hubei, and Huaxi (Wu H. et al., 2017; Tang et al., 2019; Wang M. Y. et al., 2019); *Aspergillus, Saccharomycetales, Streptomyces, Bacillus, Enterococcus*, and *Weissella* in Korean *Nuruk* (Bal et al., 2017); and *Weissella, Pediococcus, Lactobacillus, Saccharomyces, Saccharomycopsis*, and *Rhizopus* in Cambodia *Dombea* (Sokny et al., 2018). However, species may contain similar hypervariable regions, or the specific fragments that distinguish them may be outside the amplicon region, thereby creating PCR bias (Illeghems et al., 2012; Melanie et al., 2015). Compared with amplicon sequencing, metagenomic sequencing, which randomly generates small microbial gene fragments, reportedly offers an advantage for species identification (Liu et al., 2020) and may provide additional insights into the metabolic potential of the microbiome at the genetic and functional levels (Walsh et al., 2017). Indeed metagenomic sequencing has been widely applied for the identification of functional microorganisms in fermented foods, including wine (Liu et al., 2019), vinegar (Wu L. et al., 2017), cheese (Escobar-Zepeda et al., 2016), pickles (Jung et al., 2011), cocoa beans (Agyirifo et al., 2019), and fermented sausages (Ferrocino et al., 2017). Furthermore, metabolomics analysis based on gas chromatography (GC), mass spectrometry (MS), high-performance liquid chromatography (HPLC), and nuclear magnetic resonance revealed the metabolite profiles of *jiuqu* (Tang et al., 2019; Wang N. et al., 2019) and Baijiu (Huo et al., 2020; Jia et al., 2020; Liu and Miao, 2020). However, former studies on *Xiaoqu* have primarily focused on microorganisms and volatile flavors (Liu and Sun, 2018; Tang et al., 2019; Chen C. et al., 2020), whereas the correlation between microorganisms and non-volatile metabolites requires further investigation.

Therefore, herein, we used gas chromatography time-of-flight mass spectrometry (GC-TOF-MS)-based metabolomics and metagenomic approaches to analyze the distribution and metabolic potential of the microbial community, and non-volatile metabolites of three typical traditional *Xiaoqu* originating in Guizhou. Additionally, we investigated potential correlations between major microbiota with significantly different metabolites (SDMs) and physicochemical indexes. Finally, we evaluated the relationship between substrate decomposition and SDM formation in the microbial community using metabolic network analysis. Our results might provide a theoretical basis for the development of a standardized production procedure for *Xiaoqu*.

## Materials and Methods

### Sample Collection

Nine *Xiaoqu* samples were collected in August 2019 from three representative *Xiaoqu*-producing distilleries located in Huishui (HS), Anshun (AS), and Kaili (KL) in the Guizhou province, China. Samples were collected from the upper, middle, and bottom layers of the relevant *Xiaoqu* storeroom. From each layer, individual 40-g samples were collected from five separate points in the middle or surrounding edges (Supplementary Figure 1). All samples collected from each point in all three layers were then evenly combined into one mixture, from which a 150-g sample was collected using quartile method to eliminate sampling error. This process was repeated three times for each distillery. Samples were immediately transported to the laboratory, placed in sterile bags, and stored at −80°C for further analyses.

### Determination of Physical and Chemical Properties

The pH, moisture, water activity (a_w_), as well as the saccharifying, fermenting, esterifying, and liquefying power of *Xiaoqu* samples were determined according to general methods of analysis for *Daqu* (Ministry of Light Industry of China, 2011). All experiments were performed in triplicate.

### Genomic DNA Extraction

*Xiaoqu* sample (0.5 g) was weighed and placed in a triangle bottle containing 10 ml of sterilized phosphate buffer solution (pH 7.4), and 30 sterilized glass beads. After sealing, the bottle was shaken at 5°C for 1.5 h and stirred with a glass bar every 30 min. The mixture was then filtered using sterilized gauze (Shanghai, China). The filtrate was centrifuged at 4°C and 12,000 rpm for 10 min to obtain the precipitate. Subsequently, the PowerSoil DNA Isolation Kit (Carlsbad, California, USA) was used to extract total DNA from the precipitate. DNA was analyzed using 1% agar-gel electrophoresis and spectrophotometry (260/280 nm optical density ratio) and was stored at −80°C for further processing.

### Library Construction and High-Throughput Sequencing

A total of 1 μg of DNA per sample was used as input material to generate sequencing libraries using the NEBNext® Ultra™ DNA Library Prep Kit for Illumina (NEB Inc., Ipswich, MA, USA) following the manufacturer's recommendations, with added index codes to attribute sequences to each sample. Briefly, DNA samples were fragmented by sonication to a size of 350 bp, end-polished, A-tailed, and ligated with the full-length adaptor for Illumina sequencing and further PCR amplified. PCR products were purified (AMPure XP system) and libraries were analyzed for size distribution using the Agilent 2100 Bioanalyzer (Palo Alto, California, USA) and quantified using real-time PCR. Clustering of the index-coded samples was performed on a cBot Cluster Generation System according to the manufacturer's instructions. Library preparations were then sequenced on an Illumina PE-150 (San Diego, California, USA) and paired-end reads were generated.

### Quality Control

To obtain valid sequences for subsequent analysis, raw reads were generated for quality control according to the following steps: (1) removal of adapter sequence (parameter ILLUMINACLIP:adapters_PATH:2:30:10); (2) scanning of sequence (4 bp sliding window size); if the average quality score was <20 or 30 (99% accuracy), the subsequent sequence (parameter SLIDINGWINDOW:4:20 or 30) was removed; (3) removal of sequences with a final length <50 bp (parameter MINLEN:50).

### Taxonomic Assignment and Functional Annotation

The MetaPhlAn2^1^ (Truong et al., 2015) analysis tool was used to compare clean reads with unique species markers to determine the microflora composition. Finally, the normalized species abundance was calculated based on read number and marker length obtained from the comparison. Furthermore, using the HUMAnN2^2^ (Franzosa et al., 2018) software, clean reads were compared with the protein database (UniRef 90) based on DIAMOND^3^. Failed reads were filtered out and relative abundance of each protein was quantified. According to the corresponding relationship between the UniRef 90 ID and the KEGG (Kyoto encyclopedia of genes and genomes) database ID, the relative abundances of corresponding functions from the KEGG database were determined (Zhu et al., 2010; Kim et al., 2016).

### Extraction of Metabolites and Gas Chromatography Time-of-Flight Mass Spectrometry Analysis

A 20 ± 1 mg *Xiaoqu* sample, 500 μl of precooled extraction mixture [3:1 methanol/chloroform (v:v)], and 10 μl of internal standard substance (Adonito, 0.5 mg/ml stock solution) were added to a 2-ml PE tube and vortexed for 30 s. After homogenization for 4 min in a 45-Hz ball mill, the mixture was sonicated for 5 min in ice water. After centrifugation at 12,000 rpm and 4°C for 15 min, 100 μl of supernatant was placed in a 2-ml PE tube and vaporized in a vacuum concentrator. Subsequently, 60 μl of 20 mg/ml methoxyamination hydrochloride in pyridine solution was added to the sample and incubated at 80°C for 30 min. Next, the sample was derivatized using 80 μl of BSTFA reagent (1% templated mesoporous carbons, v/v) at 70°C for 1.5 h. All samples were analyzed using GC-TOF-MS after cooling to 25°C.

GC-TOF-MS results were obtained using an Agilent 7890 gas chromatograph (Palo Alto, California, USA) coupled with a time-of-flight mass spectrometer (St. Joseph, MI, USA) equipped with a DB-5MS capillary column (30 × 250 m inner diameter, 0.25 m film thickness; J&W Scientific, Folsom, CA, USA). Consecutively, 1-μl sample aliquots were injected in the splitless mode. Helium was used as the carrier gas, with a 3 ml min^−1^ front inlet purge flow, and a 1 ml min^−1^ gas flow rate through the column. The injection, transfer line, and ion source temperatures were 280, 280, and 250°C, respectively. Mass spectra was generated in the electron impact mode at 70 eV, using full-scan range of 50–500 m/z at a rate of 12.5 spectra per second after a solvent delay of 6.25 min.

### Metabolome Data Processing

Raw data analysis, including peak extraction, baseline adjustment, deconvolution, alignment, and integration were completed using the Chroma TOF (V 4.3x, LECO) software. The LECO-Fiehn Rtx5 database was used for identification of metabolites by matching the mass spectrum and retention index.

### Metabolic Profile Associated With SDMs

The KEGG database, MetaboAnalyst database, and literature data were used to collect and collate information on the SDM formation pathways and associated catalytic enzymes. The metabolic network of SDMs and microorganisms was established through gene comparisons with metagenomics sequencing data.

### Multivariate Statistical Analysis

All statistical analyses were performed using SPSS 25.0 software (IBM Inc., Chicago, IL, USA). Significant differences were determined using one-way analysis of variance (ANOVA), with *p* < 0.05 considered statistically significant. Principal component analysis (PCA) and orthogonal partial least squares discriminant analysis (OPLS-DA) were performed to analyze the GC-TOF-MS dataset using SIMCA 14.1 software (Umetrics, Umea, Sweden). Meanwhile, to avoid overfitting the model and to evaluate the model feasibility, the OPLS-DA replacement test was carried out. Based on the OPLS-DA model and ANOVA, the variable importance in projection (VIP) and *p* values were calculated and SDMs (VIP > 1, *p* < 0.05) were identified. The peak area of SDMs was visualized using the R software (version 3.6.1, Cambridge, Massachusetts, USA) and pheatmap package. Pathway and enrichment analyses were conducted by MetaboAnalyst 4.0^4^. The microbial taxonomic data were primarily analyzed using Origin (version 2019b, Origin Lab Inc., Hampton, MS, USA), and R software with the ggplot2 package was used for analyzing gene data related to KEGG pathways. Correlations between representative microorganisms with physicochemical properties and SDMs were calculated using the Spearman's rank correlation, and visualized via heatmap using R 3.6.1. Finally, integrated metabolomics and metagenomics datasets were used to generate network pathways for microbial metabolism and metabolite formation using the MetaboAnalyst 4.0^4^ and KEGG pathway^5^ databases. Each sample was verified in triplicate.

## Results and Discussion

### Analyses of Fermentation Characteristics

In general, the key factors for evaluating the quality and fermentation performances of *Xiaoqu* are its physicochemical properties (Zhang et al., 2012). Nevertheless, different process parameters and geographic regions could variably influence the physicochemical properties of *jiuqu* and thus are of vital significance (Tang et al., 2019). The various physiochemical properties for all examined samples are presented in Table 1. The moisture content of *jiuqu* must remain at ~13% to facilitate storage (Yan et al., 2015). However, we observed that the moisture contents of the three *Xiaoqu* samples were below 13%. Among these, AS *Xiaoqu* had a significantly higher moisture content, compared to the others (*p* < 0.05).

**Table 1:** Differences in physicochemical properties of three traditional *Xiaoqu* samples.

**Sample ID^A^**	**Acidity (g/kg)**	**Water activity (a_**w**_)**	**Moisture (%)**	**Fermenting power (g/1 g · 72 h)**	**Esterifying power (mg/50 g · 7 days)**	**Saccharifying power (mg/g · h)**	**Liquefying power (g/g · h)**
AS	0.38 ± 0.01^c^	0.59 ± 0.00^b^	10.7 ± 0.12^a^	1.31 ± 0.01^b^	52.04 ± 1.42^b^	952.27 ± 1.64^b^	0.96 ± 0.12^b^
HS	0.79 ± 0.01^a^	0.48 ± 0.01^c^	9.00 ± 0.07^b^	1.02 ± 0.06^c^	11.91 ± 0.67^c^	847.07 ± 1.32^c^	0.84 ± 0.02^c^
KL	0.52 ± 0.00^b^	0.63 ± 0.01^a^	6.33 ± 0.14^c^	3.23 ± 0.04^a^	114.07 ± 0.41^a^	1508.80 ± 1.31^a^	1.12 ± 0.03^a^

A *AS, Anshun; HS, Huishui; KL, Kaili. Values are presented as mean ± standard error (n = 3)*.

a−c *Different letters in the same row represent significant differences (P < 0.05)*.

The water activity, fermenting power, saccharifying power, esterifying power, and liquefying power in KL *Xiaoqu* were significantly higher than those of other samples, with the lowest values observed in AS *Xiaoqu* (*p* < 0.05). Fermentability is positively correlated with the ability to convert fermentable sugars into ethanol (Fan et al., 2018). The saccharification power refers to the ability to convert starch in *jiuqu* raw materials into sugars (Zheng et al., 2012). Meanwhile, the esterification power of *jiuqu* has been strongly linked to that of ester compounds in Baijiu (Xiong et al., 2014). Additionally, the order of acidity was HS (0.38 ± 0.01 mmol g^−1^) > KL (0.52 ± 0.00 mmol g^−1^) > AS (0.38 ± 0.01 mmol g^−1^). Currently, acidity is not only considered an important index to evaluate the degree of *jiuqu* maturity, but also an objective criteria used in *jiuqu* preparation (Yan et al., 2016).

### Overview of Metagenomic Data

A total of 227,943,215 raw reads, averaging 7.60 Gb in size and 150 bp in length, were generated from DNA extracted from nine *Xiaoqu* samples (Supplementary Table 1). We noted that the sequence GC content (%) in the datasets ranged from 36 to 41%, reflecting the predominance of microbiota with low GC content in samples (Briggs et al., 2012). After performing quality control, the percentage of high-quality reads was 95.13, 95.37, and 95.05% in AS, HS, and KL samples, respectively. Moreover, the average clean data size was 7.23 Gb, accounting for 95.18% of the original data, with the proportion of bases having a quality score higher than 20 and 30 being >98 and >95%, respectively.

### Taxonomic Analysis

**Figure 1:**
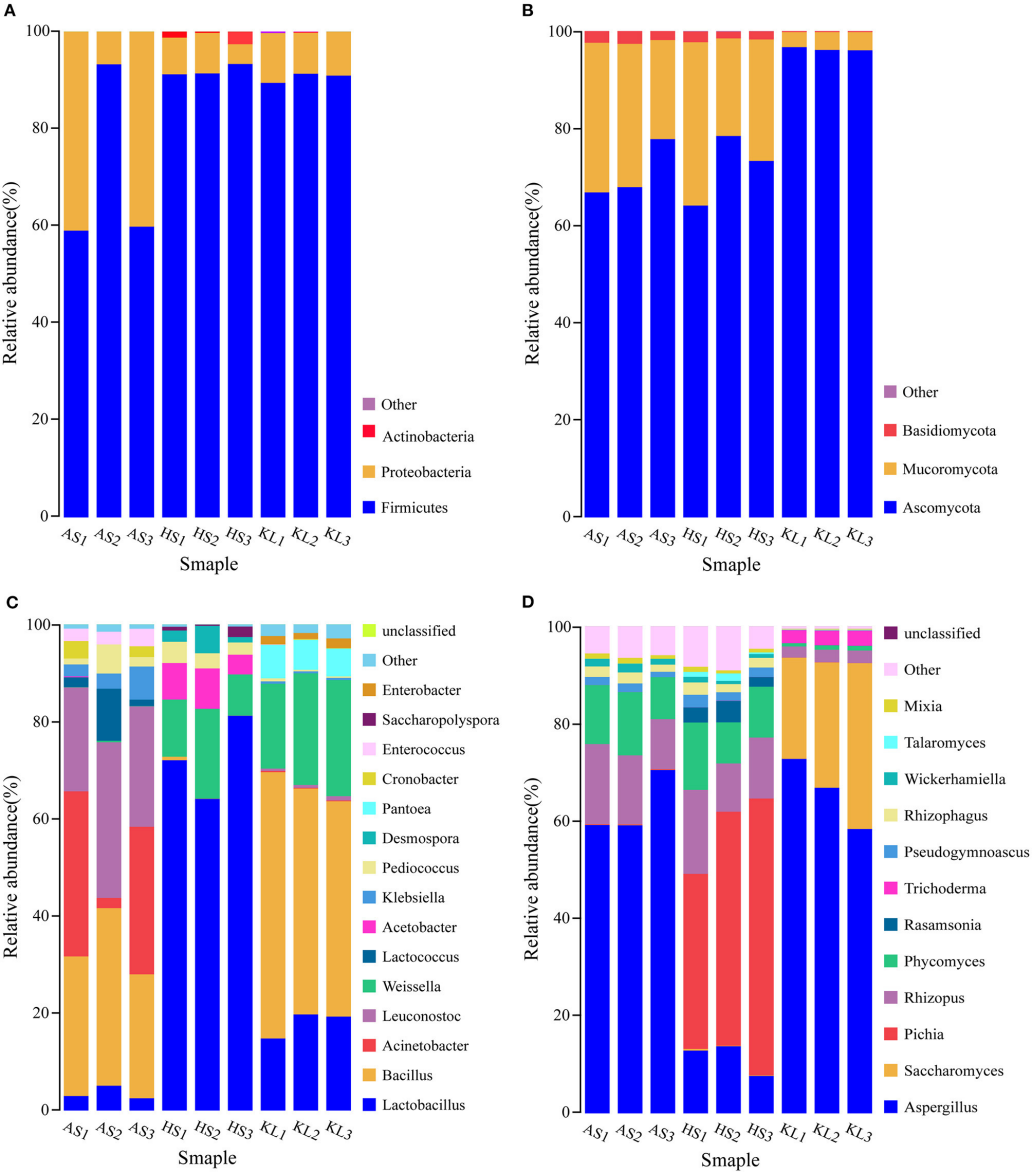
Relative abundance of bacteria **(A)** and fungi **(B)** at the phylum level and of bacteria **(C)** and fungi **(D)** at the genus level in *Xiaoqu* samples. AS, HS, and KL indicate traditional *Xiaoqu* in Guizhou: AS, Anshun; HS, Huishui; KL, Kaili.

Based on the annotation information of MetaPhlAn2, reads were assigned to different phyla and genera. At the phylum level, three dominant bacterial phyla and three dominant fungal phyla were identified in the nine *Xiaoqu* samples, including Firmicutes (84.41%), Proteobacteria (15.06%), and Actinobacteria (0.49%) and Ascomycota (79.70%), Mucoromycota (18.85%), and Basidiomycota (1.42%), respectively. The abundances of Firmicutes and Proteobacteria were 70.71, 91.97, and 90.56% and 29.29, 6.65, and 9.24% in AS, HS, and KL, respectively, whereas Actinobacteria was only detected in HS with an abundance of 1.39% (Figure 1A). Additionally, the abundances of Ascomycota and Mucoromycota were 70.85, 71.96, and 96.30% and 26.86, 26.20, and 3.49% in AS, HS, and KL, respectively (Figure 1B). However, Basidiomycota was predominantly dominant in AS (2.28%) and HS (1.77%; Figure 1B). Firmicutes, Proteobacteria, and Actinobacteria were previously identified as the dominant bacteria in *Xiaoqu* (Wu H. et al., 2017), *Xiaoqu jiu* (Dong et al., 2020), and strong-flavored Baijiu fermentation ecosystems (Zou et al., 2018). Meanwhile, Ascomycota, Mucoromycota, and Basidiomycota occupy a major position in the brewing process of black glutinous rice wine (Zhao et al., 2020), Indian dry starters (Anupma and Tamang, 2020), and strong-flavor Baijiu Daqu (Guan et al., 2015). It was, therefore, suggested that Firmicutes, Proteobacteria, Actinobacteria, Ascomycota, Mucoromycota, and Basidiomycota play a vital role in the quality and flavor of traditional *jiuqu* and fermented wine.

A total of 468 bacteria genera and 143 fungi genera were detected and classified. Figure 1C illustrates the overview of 15 bacteria genera with relative abundance >0.01 from the nine *Xiaoqu* samples, and their composition, which accounts for 98.73% of the total abundance, at the bacteria genus level. The dominant bacterial genera (>0.1 relative abundance) were *Bacillus, Lactobacillus, Weissella, Leuconostoc*, and *Acinetobacter*, which belong to Firmicutes and Proteobacteria phyla. More specifically, *Bacillus* had a dominant abundance in AS (30.23%) and KL (48.50%), whereas its abundance was only 0.27% in HS. This genus secretes various hydrolases (including amylases, proteases, lipases, etc.) and also produces a wide range of volatile compounds, including pyrazine, aldehydes, and ketones, during fermentation (Li H. et al., 2014; Li Z. M. et al., 2014; Wang et al., 2017; Jin et al., 2019). Moreover, the abundance of *Lactobacillus* and *Weissella* was predominant in HS (72.44 and 12.90%) and KL (17.98 and 21.43%), whereas *Leuconostoc* and *Acinetobacter* were dominant in AS, accounting for 26.05 and 22.13%, respectively. We also detected several lactic acid bacteria (LAB), including *Lactobacillus, Weissella*, and *Leuconostoc*, consistent with the results reported for Wuyi Qu and Gutian Qu, which are traditional *jiuqu* used in rice wine processing (Liu et al., 2018). LAB, as functional microorganisms, play a crucial role in the food industry of China (de Paiva et al., 2016) and reportedly produce bacteriocins through fermentation while accumulating organic acids via consumption of fermentable sugars. Hence, LAB can provide a lower pH condition that inhibits the propagation of pathogenic bacteria during the brewing process (Johan and Magnusson, 2005; Cappello et al., 2016), thereby facilitating enhanced sensory and nutritional values of the end-product (Frédéric and Luc, 2004). Similarly, *Acinetobacter* inhibits the growth of other microorganisms by acidifying the environment and producing biosurfactants, having a favorable degradation effect on nitrite (Al Atrouni et al., 2016). However, most *Acinetobacter* species are conditionally pathogenic bacteria. Thus, the AS *Xiaoqu* sample with its high abundance of *Acinetobacter* should be further investigated in the context of *Xiaoqu* production and the final liquor brewing process.

Microbial taxonomic assignment identified 12 fungal genera present in the nine *Xiaoqu* samples at abundances >0.01 (Figure 1D), which accounted for 95.45% of the total fungal genera abundance. The dominant fungi (>0.1 abundance) identified were *Aspergillus, Saccharomyces, Pichia, Rhizopus*, and *Phycomyces*, belonging to Ascomycota and Mucoromycota phyla. In particular, *Aspergillus* was dominant in AS, HS, and KL, accounting for 62.99, 11.46, and 66.03% of the abundance, respectively, whereas *Saccharomyces and Pichia* were only detected in KL and HS with an abundance of 26.87 and 46.97%, respectively. *Aspergillus* represented the main fungus while *Saccharomycopsis* and *Pichia* were the major yeast groups. As the most effective ethanol producer, *Saccharomyces* represents one of the core functional strains with strong fermentation capacity in liquor brewing (Wu Q. et al., 2012). However, *Pichia* primarily has an esterification role, which can enhance the ester aroma of liquor and contribute to the high ester and phenylethanol content in *Daqu* (Wang et al., 2011). Additionally, *Aspergillus* is a genus of functional aerobic microorganisms, which can survive under conditions of low humidity and high temperature (Ma et al., 2014). Thus, it is often detected in starters, such as Maotai flavor *Daqu* (Gan et al., 2019), Luzhou-flavor *Daqu* (He et al., 2019), sweet wine starter (Cai et al., 2018), and *Monascus* starter (Huang Z. R. et al., 2018), in which it secretes various enzymes and metabolic products (Wu et al., 2009). Additionally, the abundances of *Rhizopus* and *Phycomyces* were 13.64 and 11.26% and 13.24 and 10.90% in AS and HS, respectively. *Rhizopus*, a strong amylase producer that degrades starch during *Xiaoqu jiu* production (Tang et al., 2019; Dong et al., 2020), was previously identified as the main fungi in Wuyi Qu (Liu et al., 2018). Conclusively, the microbiota detected in these *Xiaoqu* samples demonstrated significant differences in their structure and diversity, which might be due to differences in geographical environment, raw materials, and preparation technology (Du et al., 2019).

### Distribution of Genes Associated With KEGG Pathways in *Xiaoqu*

**Figure 2:**
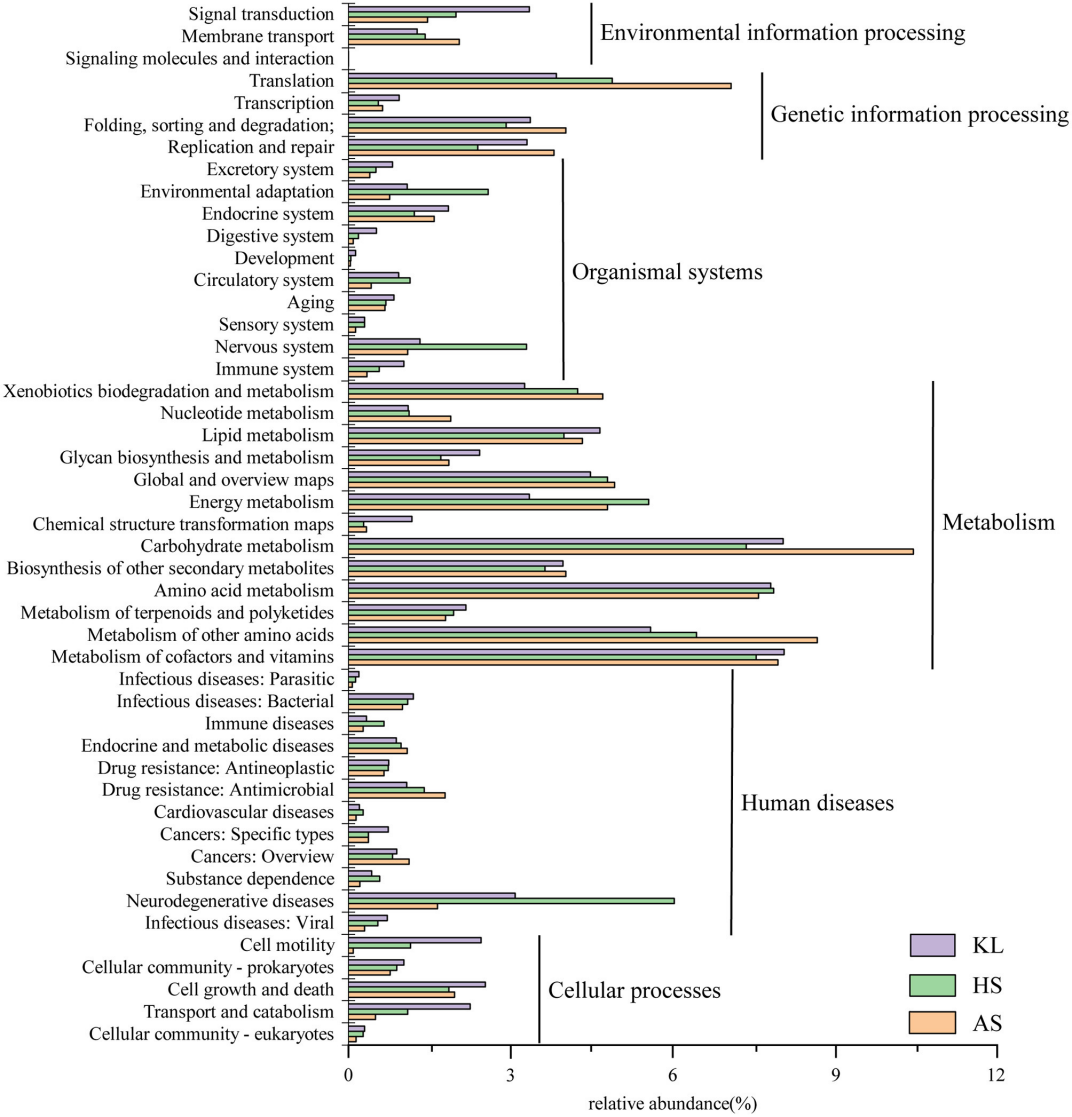
Relative abundance of functional genes predicted in KEGG pathway analysis within the microbial community in traditional *Xiaoqu* samples.

To explore the metabolic potential of the *Xiaoqu* microbiome, genes were annotated and classified using the KEGG database. Table 2 illustrates the biological metabolic pathways of functional genes, which included metabolism (58.16%), genetic information processing (12.57%), human diseases (10.92%), organismal systems (8.29%), cellular processes (5.76%), and environmental information processing (3.85%). As each category can be divided into subclasses, a total of 47 subtypes are presented in Figure 2, with metabolism of carbohydrates, cofactors and vitamins, and amino acids found to predominate (relative abundance >7%), followed by the other amino acid metabolism, transport, global and overview map, lipid metabolism, xenobiotics biodegradation and metabolism, and energy metabolism (relative abundance >4%). These results agree with those reported for Shaoxing *huang jiu*, a traditional rice wine in China (Liu et al., 2019).

**Table 2:** Relative abundance (%) of genes associated with KEGG pathways (at level 1) in three traditional *Xiaoqu* samples.

**KEGG pathway**	**AS**	**HS**	**KL**	**Total^A^**
Metabolism	18.68	20.92	18.56	58.16
Genetic information processing	3.58	5.18	3.81	12.57
Human diseases	4.53	2.89	3.50	10.92
Organismal systems	3.51	1.84	2.94	8.29
Cellular processes	1.75	1.16	2.85	5.76
Environmental information processing	1.14	1.17	1.54	3.85

*AS, Anshun; HS, Huishui; KL, Kaili*.

a *Total proportion of samples in six biometabolic pathways*.

Carbohydrates are a primary component of cell structure and energy supply and play a key role in regulation of microbial activity during fermentation. We found that the metabolism of carbohydrates accounted for the largest proportion of microbial functional genes, consistent with that reported by Xie et al. (2020) in *da-jiang*, a popular Chinese traditional fermented soybean food. Moreover, cofactors provide redox carriers for biosynthesis and decomposition reactions and are paramount in the intracellular transfer of microorganism energy (Wang et al., 2013). Therefore, regulation of cofactor abundance might be beneficial for efficient production of target metabolites (Chen et al., 2017). As small molecule organic compounds, vitamins often participate in metabolism in the form of cofactors. In addition, the metabolism of amino acids is a cardinal process in enhancing product flavor and quality (Huang X. N. et al., 2018). Meanwhile, the higher abundance of genes associated with carbohydrate, cofactor, vitamin, and amino acid metabolism indicates that starch, vitamins, and proteins serve as critical flavor precursors.

We also observed a small number of genes related to human diseases. Other researchers found similar results in fermented food, such as turbid rice wine (a Korean rice wine) (Kim et al., 2015), sweet and fermenting brewery wort (Menz et al., 2010), and traditional fermented foods in the northeast region of India (Keisam et al., 2019). According to Bolotin et al. (1999) and Olano et al. (2001), the mere existence of these undesired genes may not be represent pathogenicity of these fermented products. Further, Liu et al. (2019) and Jiang et al. (2020) found that genes related to human diseases gradually decrease during fermentation due to the inhibitory effect of LAB, yeast, ethanol, and other factors. Therefore, the safety of *Xiaoqu* is considered to be reliable.

### Analyses of Non-volatile Metabolites

**Figure 3:**
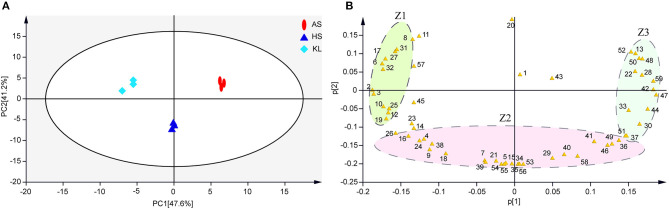
**(A,B)** Metabolite principal component analysis of samples. Colored ellipses indicate 95% confidence intervals for metabolites in each sample. AS, Anshun; HS, Huishui; KL, Kaili. 1–59, Reliable metabolite numbers in traditional *Xiaoqu* samples (Supplementary Table 2).

The results of typical GC-TOF-MS total ion chromatography of the nine samples are shown in Supplementary Figure 2, in which 797 peaks were extracted, and 795 peaks were retained after quality control. According to the degree of match (similarity ≥800) between the substances of the qualitative analysis and the substances in the standard library (Wu S. M. et al., 2012), we identified 59 reliable metabolites (Supplementary Table 2) from 795 metabolites. Subsequently, we conducted a series of multivariate pattern recognition analysis. The PCA score plot implied an obvious separation among groups and superior reliability within each group, with the variance of PC1 and PC2 being 47.6 and 41.2%, respectively (Figure 3A). Our PCA loading plot demonstrated that Z1 and Z3 mainly influenced the separation by PC 1 (47.6% of the total variance), while fumaric acid and Z2 contributed to the separation by PC 2 (41.2% of the total variance) (Figure 3B). It is obvious from the loading plot that the substances that distinguish AS, HS, and KL samples correspond to Z1, Z2, and Z3 regions, respectively. To better understand the metabolites that trigger the differences between groups, we performed OPLS-DA analysis to filter orthogonal variables that were irrelevant to categorical variables in the metabolites, and analyzed non-orthogonal variables and orthogonal variables separately to obtain more reliable model information (Trygg and Wold, 2002). Based on the results of the OPLS-DA score plots (Supplementary Figures 3A–C), samples were all within the 95% Hotelling's T-Squared Ellipse, illustrating that no outlier existed among the analyzed samples. The *R*^2^*Y* and *Q*^2^ values of the three models approached 1, indicating that they could efficiently account for the difference between samples (Lee et al., 2019). To assess the robustness and predictive ability of the OPLS-DA model, we carried out sevenfold cross-validation and permutation verification (*n* = 200; permutations experiments). The *R*^2^*Y* and *Q*^2^ values were both smaller than those of the original model (Supplementary Figures 3D–F), which was indicative of robustness and the absence of overfitting in the OPLS-DA models, thus offering an additional explanation for differences observed among AS, KL, and HS *Xiaoqu*.

**Figure 4:**
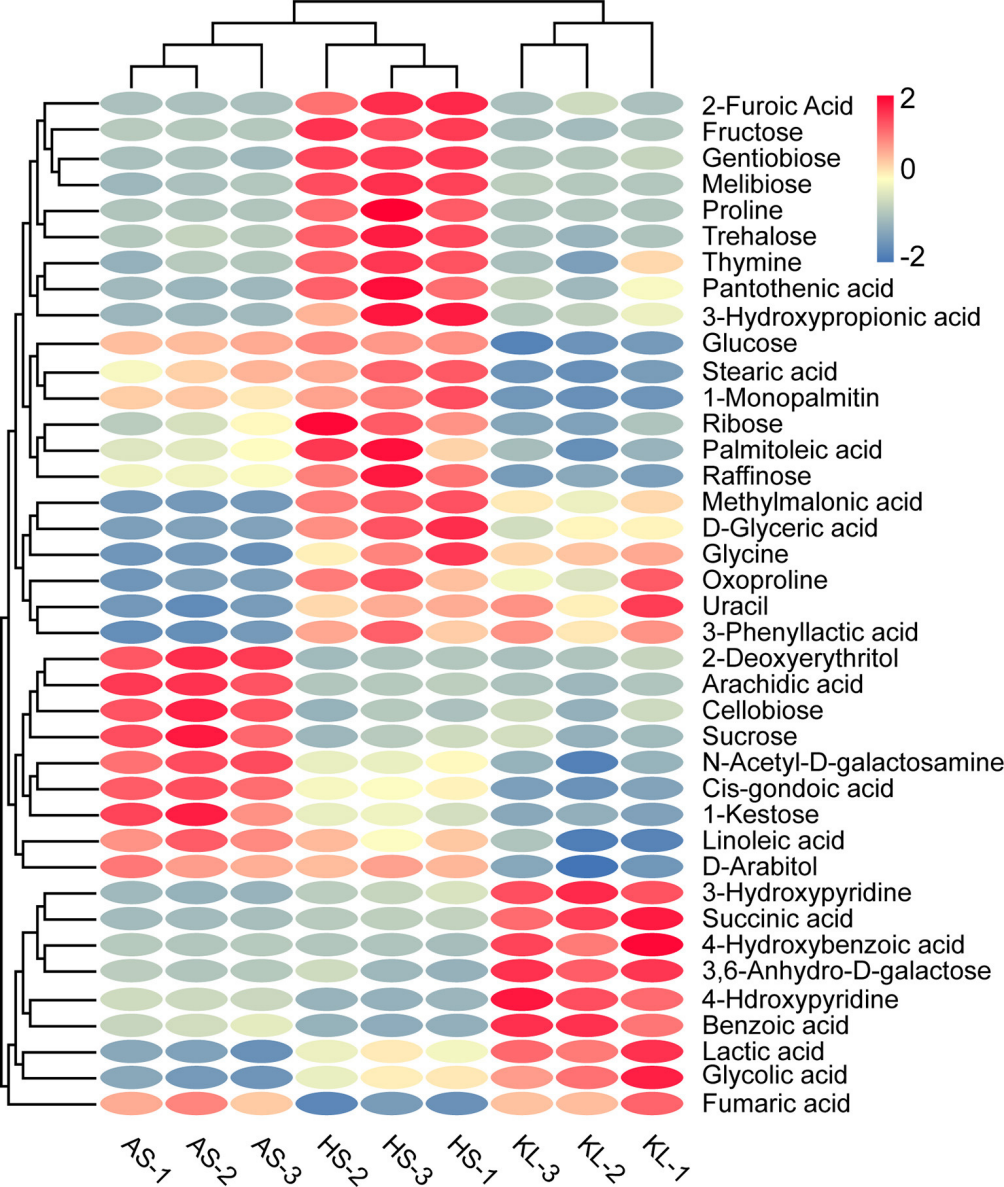
Heatmap of significantly different metabolites in combination with cluster analysis in traditional *Xiaoqu*. Dark red oval indicates higher concentration of non-volatile compounds; dark blue oval indicates lower concentration.

Note that when 59 reliable metabolites (similarity ≥800) appeared with the same name and a distinct digital number, we selected the substance with a high degree of match for future analysis (Kind et al., 2009). Meanwhile, SDMs were identified based on their VIP scores > 1.0 of OPLS-DA and *p* < 0.05 of ANOVA. In this way, we identified 39 metabolites as SDMs (Supplementary Table 3), including 12 carbohydrates, 10 organic acids, 7 fatty acids, 3 amino acids, 2 sugar alcohols, 2 nucleic acids, and 3 other compounds. We then calculated the Euclidean distance matrix using the quantitative values of SDMs, and 39 SDMs with the same characteristics were clustered though the complete linkage method, which were displayed in a heatmap to identify their group differences (Figure 4). In addition, to clarify the potential differential metabolic processes of *Xiaoqu*, we applied MetaboAnalyst 4.0 for SDM pathway enrichment. Supplementary Figure 4 demonstrates the existence of SDMs in 28 metabolic pathways. Subsequently, an impact value >0.1 was applied to determine the most relevant pathways (Chen et al., 2018), and five major metabolic pathways, namely, “starch and sucrose metabolism,” “glycine, serine, and threonine metabolism,” “glyoxylate and dicarboxylate metabolism,” “biosynthesis of pantothenate and CoA,” and “pyruvate metabolism” were identified. Interestingly, “metabolism of carbohydrates,” “metabolism of cofactors and vitamins,” and “metabolism of amino acids” mentioned in the obtained metagenomic data correspond to “starch and sucrose metabolism,” “biosynthesis of pantothenate and CoA,” and “glycine, serine, and threonine metabolism” in the metabonomics data, respectively. The high matching between the data results not only shows the feasibility of the method, but also verifies the metabolic pathway of *Xiaoqu*.

Carbohydrates and organic acids were found to be the kernel metabolites leading to differences in AS and KL, respectively. However, carbohydrates, organic acids, amino acids, and fatty acids were also found to represent differential metabolites in the clustering of integrated HS *Xiaoqu*. Carbohydrates enter the tricarboxylic acid (TCA) cycle through the glycolysis pathway for the synthesis of organic acids, amino acids, and other flavor metabolites during the fermentation process, and provide energy for microbial growth via the carbohydrate metabolism pathway (Gu et al., 2017). Herein, we observed that the SDMs included several carbohydrates in AS *Xiaoqu*, such as cellobiose, sucrose, and 1-kestose. Previous studies inferred that cellobiose was the end-product of the endo-β-glucanase gene in Luzhou-flavor *Daqu* (Ali et al., 2018), having a positive effect on extracellular β-glucosidase production (Swangkeaw et al., 2009). Additionally, we identified fructose, melibiose, trehalose, glucose, ribose, and raffinose as the major carbon compounds in HS *Xiaoqu*. Among these, trehalose has a prominent protective effect on biological cells, and thus can serve as a natural storage carbon source and a protective agent to protect biomolecular structures from destruction (Kim et al., 2011). Besides, glucose and raffinose form stable mixtures with bisulfite under certain conditions (Harbertson et al., 2013). Meanwhile, sugar reportedly exerts minimal effects on the direct sensory attributes of Baijiu as it is primarily involved in metabolism during the fermentation process, with a low content present in the final product (<2% of the total non-volatile profile) (Fang et al., 2019; Tan et al., 2019).

Organic acids are not only paramount aroma and taste substances in liquor, but also flavor precursors. The appropriate content of organic acids can make the liquor body elegant and delicate or mellow and soft with a long aftertaste (Xu et al., 2018). We found that succinic acid, lactic acid, fumaric acid, and glycolic acid represented the distinguishable SDMs in KL *Xiaoqu*. Succinic acid, a main product of the TCA cycle, is known as the “wine skeleton” (Zhang et al., 2017) and, when combined with lactic acid, provides the unique sourness and umami taste to the final product. Additionally, lactic acid is the central precursor of ethyl lactate, which is the key flavoring component in liquor (Cai et al., 2019; Liu and Miao, 2020). Meanwhile, fumaric acid improves the stability and freshness of wine and decreases the level of SO_2_ (Morata et al., 2018). Furthermore, methylmalonic acid, 2-furoic acid, 3-phenyllactic acid, and 3-hydroxypropionic acid were clustered in HS *Xiaoqu*. The decarboxylation of 2-furoic acid was confirmed as an alternative pathway in the formation of furan in heat-treated foods (Delatour et al., 2019), whereas 3-phenyllactic acid, which is widely present in fermented foods, has highly effective and broad-spectrum antimicrobial activity (Xu et al., 2020). However, we did not observe any dominant organic acids in AS *Xiaoqu*.

Among the key HS *Xiaoqu* metabolites, we identified three amino acids, namely, proline, glycine, and oxoproline. Among them, proline is reportedly beneficial for providing balance and softness in the taste of wine (Hu B. R. et al., 2020). Moreover, isoamylol, phenylethanol, and β-phenylethanol form from the amino acids leucine, phenylalanine, and phenylalanine, respectively, and are crucial flavor precursors (Chen Y. et al., 2020), contributing to the sweet, fresh, and bitter taste of wine (Procopio et al., 2015; Yin et al., 2017). Meanwhile, as major nitrogen sources for the survival of microorganisms, amino acids are closely related to the growth of microorganisms and production of metabolites (Park et al., 2013; Yang et al., 2018) and can interact with carbohydrates via the Maillard reaction to produce aromatic substances (Wang W. Y. et al., 2018). We also identified four SDMs, namely, linoleic acid, stearic acid, arachidic acid, and d-glyceric acid, as the major fatty acids in HS *Xiaoqu*. These fatty acids are involved in the composition of liquor flavor in liquor and are also associated with various health-related functions. For instance, linoleic acid and stearic acid, which are present in myrtle liqueur (Correddu et al., 2019), Maotai flavor liquor (Cai et al., 2019), and cacao liquor (Osakabe et al., 1998), have been reported to exhibit antitumor, antiobesity, antidiabetic, and anti-inflammatory properties (Jaudszus et al., 2016). Moreover, arachidic acid, a C20 saturated fatty acid, was implicated in the incidence of atherosclerosis and coronary heart disease (Rivellese et al., 2003). Generally, regarding their non-volatile profiles, the features of these samples were shown to be sufficiently different, indicating that the non-volatile components of *Xiaoqu* might be impacted by several factors, including microbial abundance, raw materials, or physical location.

### Correlation Analysis of Representative Microbiota With Physicochemical Properties and SDMs

It is generally accepted that the differences in microbiota play a major role not only in the flavor and taste, but also in the formation of metabolites. Therefore, we explored the relationship of the seven physicochemical indexes, 39 SDMs, and the top 15 genera in the relative abundance of all species in the nine *Xiaoqu* samples using Spearman's algorithm. To further clarify the associated relationships, we carried out clustering heatmap using the R software.

**Figure 5:**
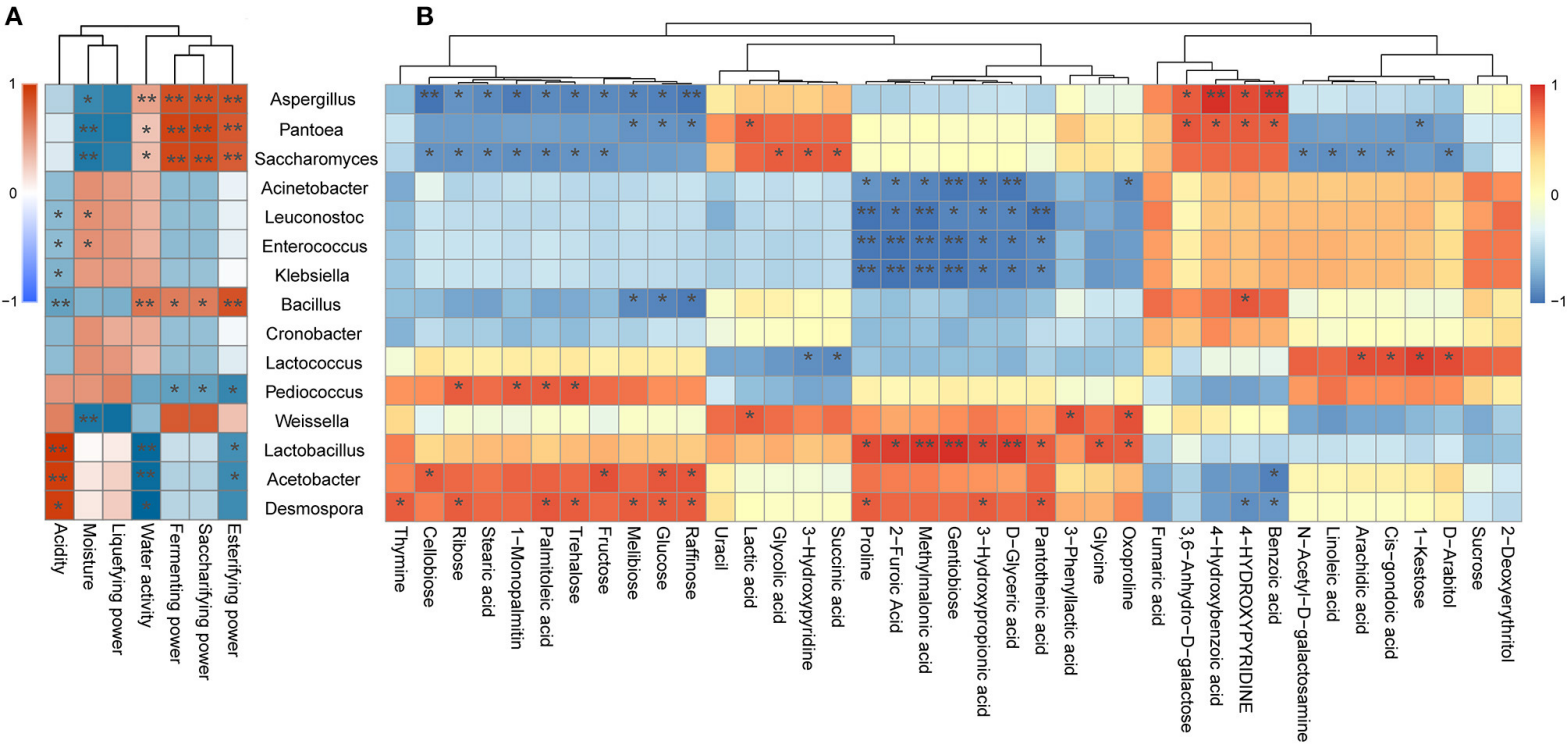
**(A)** Correlation between physicochemical properties and representative microbial taxa identified in the three traditional *Xiaoqu*. **(B)** Correlation between significantly different metabolites and representative microbial taxa identified in the three traditional *Xiaoqu*. Red represents a positive correlation; blue represents a negative correlation. *p < 0.05, **p < 0.001.

As illustrated in Figure 5, *Aspergillus* was found to be positively associated with the esterifying power, saccharifying power, fermenting power, water activity, benzoic acid, 4-hydroxybenzoic acid, 4-hydroxypyridine, and 3,6-anhydro-d-galactose, whereas it was negatively associated with seven carbohydrates, including raffinose, glucose, melibiose, fructose, trehalose, ribose, and cellobiose. Moreover, *Aspergillus* as the pivotal mold detected in *jiuqu* likely plays a key role in the saccharification, fermentation, and esterification of wine (Wu et al., 2009; Liang et al., 2016), as it secretes a variety of extracellular enzymes, including proteolytic enzymes and starch hydrolytic enzymes, thereby leading to the production of flavor metabolites and flavor precursors (Wicklow et al., 2007; Jing et al., 2014). These results support the conclusion that *Aspergillus* promotes formation of hydrolase, sugar, and acid in *Xiaoqu*. Furthermore, *Saccharomyces* was positively correlated with the esterifying power, saccharifying power, fermenting power, succinic acid, and glycolic acid, whereas it was negatively associated with fructose, trehalose, ribose, cellobiose, palmitoleic acid, stearic acid, and 1-monopalmitin. Yeast can convert carbohydrates into ethanol, producing a series of key flavor compounds, such as acids, esters, and higher alcohols (Li H. et al., 2014; Li Z. M. et al., 2014; Liu et al., 2017; Fan et al., 2019). Therefore, it was not surprising that the *Saccharomyces* genus exhibited this type of correlation (Figure 5). Note that *Lactobacillus* exhibited a significant and positive correlation with acidity, methylmalonic acid, d-glyceric acid, 2-furoic acid, methylmalonic acid, pantothenic acid, gentiobiose, oxoproline, glycine, and proline (Figure 5; *p* < 0.05). Moreover, LAB can produce a large amount of organic acids, amino acids, and fatty acids (Ho et al., 2014; Huang X. N. et al., 2018; Jin et al., 2019; Zang et al., 2020), which was supported by the current study results. Moreover, by reducing acidity, LAB can also inhibit the growth and reproduction of pathogenic bacteria during the fermentation system (Cappello et al., 2016; Huang et al., 2019). In our study, *Acetobacter* was positively correlated with acidity (Figure 5A), which was consistent with previous reports that described *Acetobacter* as one of the major contributors to acidity in the Shanxi aged fermented vinegar and Luzhou-flavor liquor pit mud (Li et al., 2016; Jiang et al., 2019). *Acetobacter* was also found to be positively correlated with four carbohydrates, namely, cellobiose, fructose, raffinose, and glucose (Figure 5B). In fact, *Acetobacter* contains amylase activity and can hydrolyze starch from raw materials into sugar compounds (Ye et al., 2019). Besides, *Bacillus* is significantly positively correlated with esterifying power, saccharifying power, fermenting power, and water activity (Figure 5A). A previous study found that *Bacillus* could degrade cellulose, starch, and protein into substances available for subsequent fermentation and promote the production of alcohols (Tang et al., 2019), which agrees with our results.

It is worth noting that *Acinetobacter, Enterococcus*, and *Klebsiella* showed significant negative correlations with most SDMs (Figure 5; *P* < 0.05). Members of the *Acinetobacter, Enterococcus*, and *Klebsiella* genera are recognized as synthetic acetoin bacteria during fermentation (Molinari et al., 2003; Chen et al., 2010), thus posing a health risk to consumers (Liu and Sun, 2018). Fortunately, it was demonstrated that *Weissella* exerts strong antagonism against these undesirable microorganisms (Supplementary Table 4), which was supported by the conclusion that *Weissella* enhances the production of acids and alcohols to inhibit the growth of pathogenic bacteria (Chen C. et al., 2020). Moreover, pathogenic microorganisms only represent a small portion of the microbial community in the early steps of black glutinous rice wine fermentation (Jiang et al., 2020). Likewise, Liu et al. (2019) and Mu et al. (2020) reported that these undesirable microorganisms disappeared during fermentation. Hence, further research is required to determine the mechanism by which pathogenic bacteria become destroyed. Besides, we find that *Pantoea* and *Desmospora* exhibited significant and positive correlation with most SDMs (Figure 5; *P* < 0.05). Although the roles of members from these genera in the fermentation of *Xiaoqu* wine have not yet been fully elucidated, they have been studied extensively in other fields. For example, the genus *Pantoea*, belonging to the family *Enterobacteriaceae*, represents a genus comprising common endophytes of rice (Megías et al., 2016). Members from this genus are commonly used as biological control agents due to their excellent antimicrobial production capacity (Walterson et al., 2014). Meanwhile, *Desmospora* may represent a new member of the DTEase family of enzymes with highest substrate specificity toward d-psicose (Zhang et al., 2013). This genus was isolated from a deep-sea sediment and was recently detected in traditional fermentation of Chinese Fen-Maotai-flavored liquor in pottery jars (Zhang et al., 2015; Hu X. X. et al., 2020).

### Construction of the Metabolic Network

**Figure 6:**
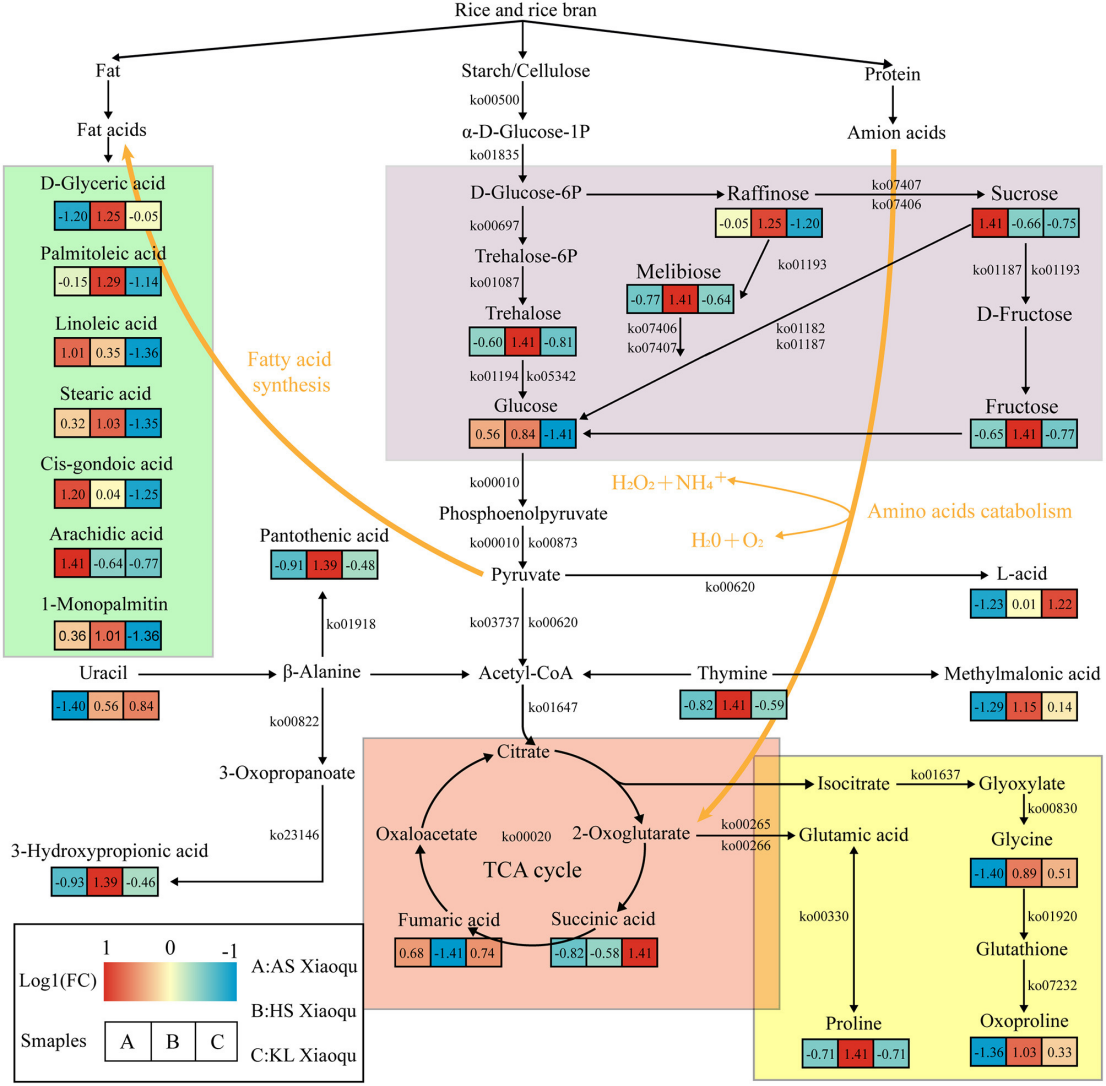
Predicted metabolic network for flavor formation in *Xiaoqu*. Beneath each metabolite, the color gradient and their values indicate the log1 (fold change) with respect to *Xiaoqu*; red and blue represent up- and downregulated metabolites, respectively. Carbohydrate metabolism, TCA cycle, amino acid metabolism, as well as biosynthesis and degradation of fatty acids are shown in purple, red, yellow, and green, respectively.

To better understand the relationship between microorganisms and metabolites, we used metabolomics, metagenomics, and related network data to predict the metabolic network associated with substrate decomposition and SDM formation in Guizhou *Xiaoqu* (Figure 6). Starch, cellulose, protein, and fat were the raw materials of *Xiaoqu* and the main substrates for metabolite formation. Meanwhile, melibiose in raw materials, as well as raffinose, sucrose, fructose, and glucose degraded from starch and cellulose in rice and rice bran by microorganisms, serve as alternative carbon sources for the production of organic acids, amino acids, and fatty acids Wu L. et al., 2017; Jin et al., 2019. Pyruvate and acetyl-CoA are further produced by microbial degradation as central compounds of the metabolic network. Acetyl-CoA participates in the TCA cycle and fatty acid biosynthesis. Moreover, organic acids, including succinic and fumaric acid, that are generated through the TCA cycle are further converted into proline, glycine, and oxoproline (He et al., 2019), all of which were abundant in HS and KL *Xiaoqu*, and which may serve as nitrogen sources for microbial growth during fermentation and as flavor precursors (Fairbairn et al., 2017). The nucleic acids in the raw materials of *Xiaoqu*, including uracil and thymine, can also be used as a nitrogen source during the fermentation of *Xiaoqu*. Specifically, uracil can be degraded to acetyl-CoA, 3-hydroxypropionic acid, and pantothenic acid during the fermentation of HS *Xiaoqu* (Zhu et al., 2020), whereas thymine can be degraded to acetyl-CoA and methylmalonic acid in KL *Xiaoqu*. Besides, pyruvate can be degraded to lactic acid in KL *Xiaoqu* (Ai et al., 2019). Fatty acids, including d-glyceric acid, palmitoleic acid, linoleic acid, stearic acid, cis-gondoic acid, arachidic acid, and 1-monopalmitin, produced by acetyl-CoA through the fatty acid biosynthesis pathway or fat degradation, were also identified as major compounds in AS and HS *Xiaoqu* (Mu et al., 2020). In summary, the metabolism of carbohydrates and TCA cycle appear to play a paramount role in the formation of non-volatile compounds in *Xiaoqu*.

## Conclusion

Using metagenomics and metabolomics analyses, we investigated potential correlations between the microbial community and metabolites produced in traditional *Xiaoqu* collected in Guizhou, China. Our metabolomics data results were highly consistent with those of the metagenomics data. Eight microbial genera, namely, *Aspergillus, Saccharomyces, Lactobacillus, Acetobacter, Weissella, Pantoea, Desmospora*, and *Bacillus*, were positively correlated with physicochemical indexes and SDMs. Besides, we established the flavor metabolic network in the microbiota of Guizhou *Xiaoqu* and revealed the decomposition profile for substrates and formation of SDMs in metabolic pathways. Further isolation and identification of functional strains in *Xiaoqu* is warranted, including characterization of the functions and fermentation mechanisms of these strains using multiomics methods, such as macro transcriptomics and metaproteomics. These findings provide insights for the use of specific functional strains to provide biological enhancement, as well as improved yield stability and quality in *Xiaoqu* wine production.

## Data Availability Statement

The datasets presented in this study can be found in online repositories. The names of the repository/repositories and accession number(s) can be found in the article/Supplementary Material.

## Author Contributions

CZ and WS contributed to the experimental design, performed the statistical analysis, and wrote the manuscript. CZ, WS, YuM, YiM, and LJ contributed to manuscript revision, read, and approved the submitted version. All authors contributed to the article and approved the submitted version.

## Conflict of Interest

The authors declare that the research was conducted in the absence of any commercial or financial relationships that could be construed as a potential conflict of interest.

## Footnotes

^1^http://huttenhower.sph.harvard.edu/metaphlan2

^2^http://huttenhower.sph.harvard.edu/humann2

^3^http://ab.inf.uni-tuebingen.de/software/diamond/

^4^www.metaboanalyst.ca

^5^egg.jp/kegg/pathway.html
