# Supplementary material for: Integrative Metagenomics–Metabolomics for Analyzing the Relationship Between Microorganisms and Non-volatile Profiles of Traditional Xiaoqu
Source: Front Microbiol. 2021 Feb 1;11:617030. doi: 10.3389/fmicb.2020.617030 (PMC7882485; doi:10.3389/fmicb.2020.617030)
Supplement: Supplementary file 1 [file Data_Sheet_1.PDF]

frontiers

Supplementary Material

1 Supplementary Figures and Tables

1.1 Supplementary Figures

Supplementary Figure S1. *Xiaoqu* samples collection process.

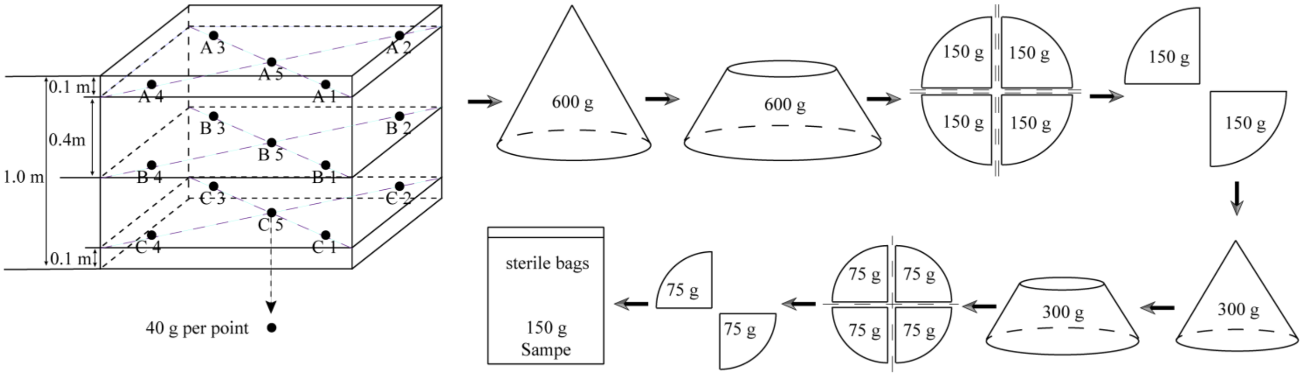

Supplementary Figure S2. The GC-TOF-MS typical total ion chromatograms (TICs) of traditional *Xiaoqu* samples in Guizhou.

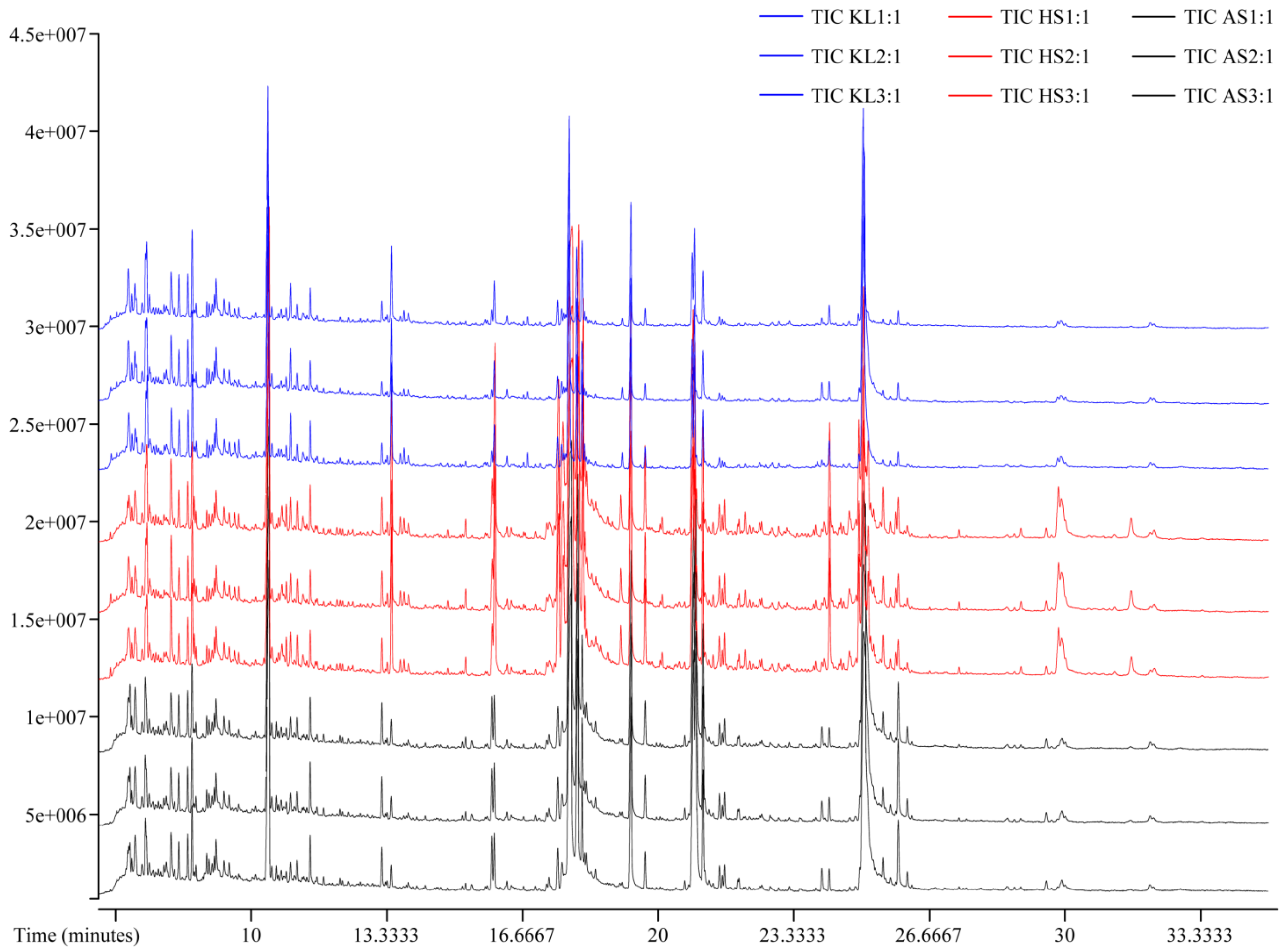

Supplementary Figure S3**.** Orthogonal partial minimum discriminant analysis scores of the OPLS-DA model (A, D-AS vs HS; B, E-AS vs KL; C, F-HS vs KL) and cross-validation.

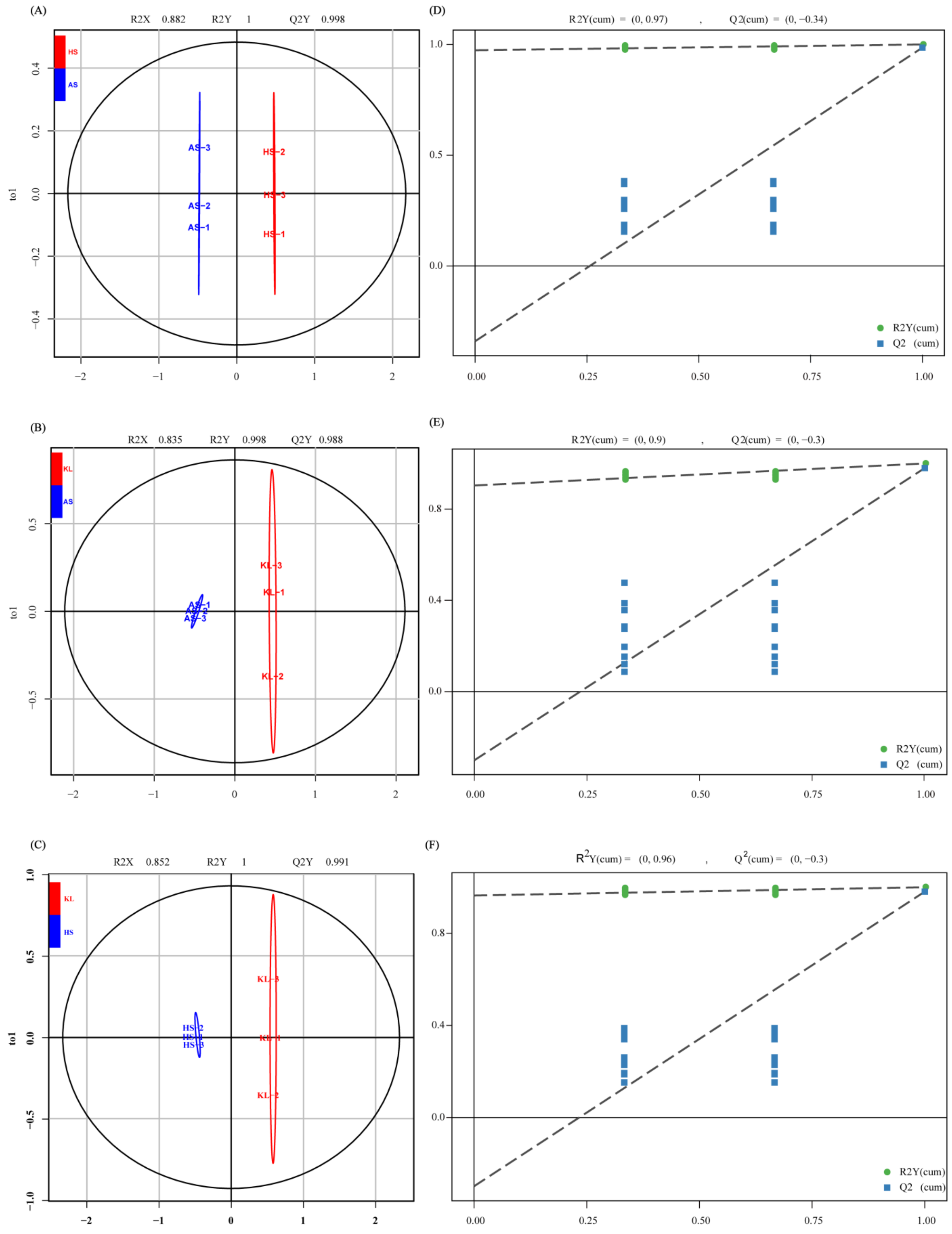

Supplementary Figure S4. Enriched bubble chart of differential metabolite pathways (bubble size is proportional to the degree of influence of each pathway; bubble color indicates the significant degree of influence, from the highest (red) to the lowest (white)).

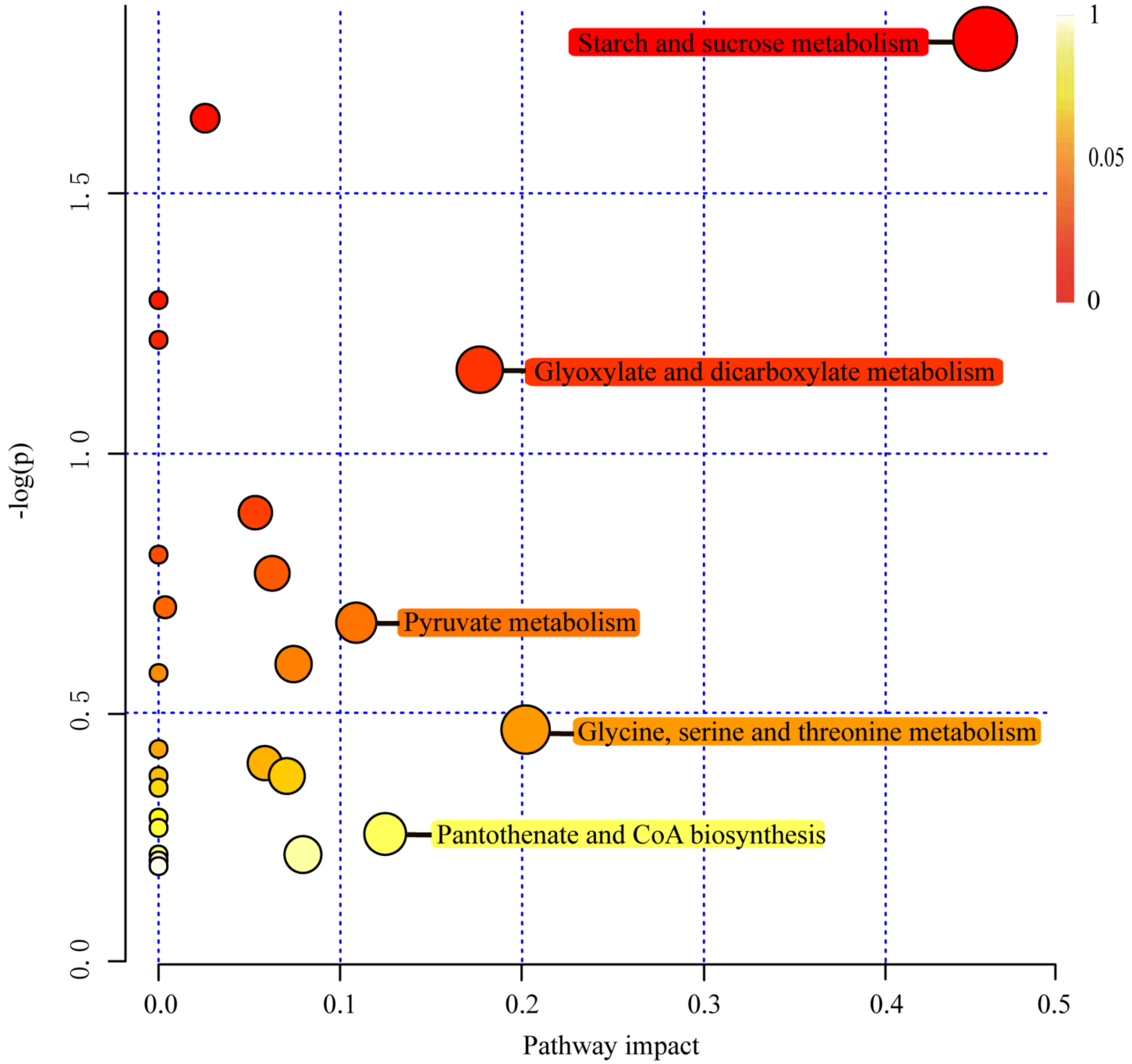

1.2 Supplementary Tables

Supplementary Table S1. Statistics of sequencing and bioinformatics analysis.

Smple ID [a]	InsertSize (bp)	SeqStrategy	RawReads	Raw Base(GB)	%GC	Raw Q20(%)	Raw Q30(%)	Clean Reads	Cleaned (%)	Clean Q20(%)	Clean Q30(%)
AS1	350	(150:150)	26042595	7.81	36	97.97	94.08	24791440	95.20	98.91	95.56
AS2	350	(150:150)	22348520	6.70	36	97.94	93.99	21322888	95.41	98.87	95.46
AS3	350	(150:150)	22260835	6.68	36	97.68	93.54	21099976	94.79	98.84	95.37
HS1	350	(150:150)	29188580	8.76	38	98.03	94.16	27828756	95.34	98.90	95.57
HS2	350	(150:150)	28837018	8.65	41	98.17	94.44	27527733	95.46	98.96	95.74
HS3	350	(150:150)	28400932	8.52	40	98.08	94.28	27070144	95.31	98.93	95.66
KL1	350	(150:150)	24160438	7.25	38	97.90	94.08	23119875	95.69	98.92	95.69
KL2	350	(150:150)	23556471	7.07	37	98.07	94.18	22507377	95.55	98.89	95.50
KL3	350	(150:150)	23147826	6.94	37	98.06	94.17	21730849	93.88	98.94	95.63

[a] AS1, AS2, AS3, HS1, HS2, HS3, KL1, KL2, and KL3 indicates the traditional *Xiaoqu* in Guizhou: AS1, AS2, and AS3) Anshun; HS1, HS2, and HS3) Huishui; KL1, KL2, and KL3) Kaili.

Supplementary Table S2. Reliable metabolites in traditional *Xiaoqu* samples (similarity ≥ 800).

NO. [a]	Metabolites	Similarly	RT (min)	CAS No.	Formula	Identified ion (m/z)
1	2-Hydroxypyridine	899	7.14	142-08-5	C_5H_5NO	152
2	Lactic acid	957	7.43	50-21-5	$C_3H_6O_3$	117
3	Glycolic acid	824	7.65	79-14-1	$C_2H_4O_3$	66
4	Alanine 1	924	8.02	56-41-7	$C_3H_7NO_2$	116
5	2-Furoic Acid	854	8.52	88-14-2	$C_5H_4O_3$	125
6	3-Hydroxypyridine	861	8.58	109-00-2	C_5H_5NO	152
7	3-Hydroxypropionic acid 1	924	8.60	503-66-2	$C_3H_6O_3$	177
8	4-HYDROXYPYRIDINE	822	8.81	626-64-2	C_5H_5NO	152
9	Methylmalonic acid	830	9.43	516-05-2	$C_4H_6O_4$	233
10	Valine	863	9.60	72-18-4	$C_5H_{11}NO_2$	144
11	Benzoic acid	862	10.12	65-85-0	$C_7H_6O_2$	179
12	Ethanolamine	868	10.31	141-43-5	C_2H_7NO	174
13	2-Deoxyerythritol	906	10.61	3068-00-6	$C_4H_{10}O_3$	117
14	Isoleucine	882	10.68	131598-62-4	$C_6H_{13}NO_2$	158
15	Proline	869	10.75	344-25-2	$C_5H_9NO_2$	142
16	Glycine 2	947	10.86	56-40-6	$C_2H_5NO_2$	174
17	Succinic acid	937	10.96	110-15-6	$C_4H_6O_4$	147
18	D-Glyceric acid	910	11.15	6000-40-4	$C_3H_6O_4$	189
19	Uracil	844	11.28	66-22-8	$C_4H_4N_2O_2$	241
20	Fumaric acid	947	11.45	110-17-8	$C_4H_4O_4$	245

21	Thymine	864	12.10	65-71-4	$C_5H_6N_2O_2$	255
22	L-Malic acid	928	13.21	97-67-6	$C_4H_6O_5$	73
23	Threitol	912	13.34	7493-90-5	$C_4H_{10}O_4$	73
24	Oxoproline	901	13.66	98-79-3	$C_5H_7NO_3$	156
25	4-aminobutyric acid 1	908	13.75	20791	$C_4H_9NO_2$	174
26	3-Phenyllactic acid	821	14.40	828-01-3	$C_9H_{10}O_3$	193
27	4-Hydroxybenzoic acid	882	14.94	99-96-7	$C_7H_6O_3$	223
28	Xylose 1	897	15.19	6763-34-4	$C_5H_{10}O_5$	103
29	Ribose	922	15.27	24259-59-4	$C_5H_{10}O_5$	103
30	D-Arabitol	942	15.92	488-82-4	$C_5H_{12}O_5$	217
31	3,6-Anhydro-D-galactose 3	895	16.29	14122-18-0	$C_6H_{10}O_5$	231
32	3,6-Anhydro-D-galactose 4	873	16.69	14122-18-0	$C_6H_{10}O_5$	231
33	Citric acid	851	16.99	5949-29-1	$C_6H_8O_7$	273
34	Fructose 1	961	17.54	57-48-7	$C_6H_{12}O_6$	103
35	Fructose 2	896	17.64	57-48-7	$C_6H_{12}O_6$	307
36	Glucose 1	852	17.85	50-99-7	$C_6H_{12}O_6$	160
37	Glucose 2	869	18.02	50-99-7	$C_6H_{12}O_6$	103
38	Sorbitol	954	18.14	50-70-4	$C_6H_{14}O_6$	307
39	Pantothenic acid	808	18.76	79-83-4	$C_9H_{17}NO_5$	201
40	Palmitoleic acid	895	19.11	373-49-9	$C_{16}H_{30}O_2$	117
41	Palmitic acid	954	19.33	21096	$C_{16}H_{32}O_2$	132
42	N-Acetyl-D-galactosamine 1	888	19.56	14215-68-0	$C_8H_{15}NO_6$	87
43	Myo-inositol	925	19.69	87-89-8	$C_6H_{12}O_6$	217

44	Linoleic acid	926	20.85	60-33-3	$C_{18}H_{32}O_2$	337
45	Elaidic acid	920	20.90	112-79-8	$C_{18}H_{34}O_2$	339
46	Stearic acid	950	21.11	57-11-4	$C_{18}H_{36}O_2$	132
47	Cis-gondoic acid	811	22.56	5561-99-9	$C_{20}H_{38}O_2$	145
48	Arachidic acid	893	22.75	506-30-9	$C_{20}H_{40}O_2$	132
49	1-Monopalmitin	874	23.87	19670-51-0	$C_{19}H_{38}O_4$	371
50	Sucrose	900	24.21	57-50-1	$C_{12}H_{22}O_{11}$	361
51	Cellobiose 1	802	24.70	528-50-7	$C_{12}H_{22}O_{11}$	204
52	Cellobiose 2	870	24.94	528-50-7	$C_{12}H_{22}O_{11}$	160
53	Trehalose	849	25.05	99-20-7	$C_{12}H_{22}O_{11}$	67
54	Gentiobiose 1	917	25.53	554-91-6	$C_{12}H_{22}O_{11}$	204
55	Melibiose 1	920	25.90	66009-10-7	$C_{12}H_{24}O_{12}$	204
56	Melibiose 2	901	26.12	66009-10-7	$C_{12}H_{24}O_{12}$	361
57	Palatinitol 2	870	26.22	64519-82-0	$C_{12}H_{24}O_{11}$	204
58	Raffinose	881	29.83	512-69-6	$C_{18}H_{32}O_{16}$	204
59	1-Kestose	850	29.92	470-69-9	$C_{18}H_{34}O_{16}$	230

[a] Numbered as in the retention time.

Supplementary Table S3. Main variables contributing to discrimination among traditional *Xiaoqu* samples.

NO. [a]	Metabolites	Similarly	RT (min)	CAS No.	Formula	Identified ion (m/z)	VIP	*P*
1	Lactic acid	957	7.43	50-21-5	$C_3H_6O_3$	117	1.03659	< 0.001
2	Glycolic acid	824	7.65	79-14-1	$C_2H_4O_3$	66	1.0283	< 0.001
3	2-Furoic Acid	854	8.52	88-14-2	$C_5H_4O_3$	125	1.06012	0.005
4	3-Hydroxypyridine	861	8.58	109-00-2	C_5H_5NO	152	1.03952	< 0.001
5	3-Hydroxypropionic acid	924	8.60	503-66-2	$C_3H_6O_3$	177	1.04328	0.029
6	4-Hydroxypyridine	822	8.81	626-64-2	C_5H_5NO	152	1.04636	< 0.001
7	Methylmalonic acid	830	9.43	516-05-2	$C_4H_6O_4$	233	1.06518	< 0.001
8	Benzoic acid	862	10.12	65-85-0	$C_7H_6O_2$	179	1.04964	< 0.001
9	2-Deoxyerythritol	906	10.61	3068-00-6	$C_4H_{10}O_3$	117	1.03498	< 0.001
10	Proline	869	10.75	344-25-2	$C_5H_9NO_2$	142	1.06742	0.006
11	Glycine	947	10.86	56-40-6	$C_2H_5NO_2$	174	1.012	0.001
12	Succinic acid	937	10.96	110-15-6	$C_4H_6O_4$	147	1.04366	0.002
13	D-Glyceric acid	910	11.15	6000-40-4	$C_3H_6O_4$	189	1.05039	0.005
14	Uracil	844	11.28	66-22-8	$C_4H_4N_2O_2$	241	1.02328	< 0.001
15	Fumaric acid	947	11.45	110-17-8	$C_4H_4O_4$	245	1.03074	< 0.001
16	Thymine	864	12.10	65-71-4	$C_5H_6N_2O_2$	255	1.03179	0.003
17	Oxoproline	901	13.66	98-79-3	$C_5H_7NO_3$	156	1.00486	0.006
18	3-Phenyllactic acid	821	14.40	828-01-3	$C_9H_{10}O_3$	193	1.03385	< 0.001
19	4-Hydroxybenzoic acid	882	14.94	99-96-7	$C_7H_6O_3$	223	1.03515	0.008

20	Ribose	922	15.27	24259-59-4	$C_5H_{10}O_5$	103	1.02085	< 0.001
21	D-Arabitol	942	15.92	488-82-4	$C_5H_{12}O_5$	217	1.0326	< 0.001
22	3,6-Anhydro-D-galactose	895	16.29	14122-18-0	$C_6H_{10}O_5$	231	1.04236	< 0.001
23	Fructose	896	17.64	57-48-7	$C_6H_{12}O_6$	307	1.07438	< 0.001
24	Glucose	869	18.02	50-99-7	$C_6H_{12}O_6$	103	1.04944	< 0.001
25	Pantothenic acid	808	18.76	79-83-4	$C_9H_{17}NO_5$	201	1.06444	0.012
26	Palmitoleic acid	895	19.11	373-49-9	$C_{16}H_{30}O_2$	117	1.00037	0.007
27	N-Acetyl-D-galactosamine	888	19.56	14215-68-0	$C_8H_{14}NO_6$	87	1.01345	0.002
28	Linoleic acid	926	20.85	60-33-3	$C_{18}H_{32}O_2$	337	1.00812	< 0.001
29	Stearic acid	950	21.11	57-11-4	$C_{18}H_{36}O_2$	132	1.04657	< 0.001
30	Cis-gondoic acid	811	22.56	5561-99-9	$C_{20}H_{38}O_2$	145	1.03569	< 0.001
31	Arachidic acid	893	22.75	506-30-9	$C_{20}H_{40}O_2$	132	1.04	< 0.001
32	1-Monopalmitin	874	23.87	19670-51-0	$C_{19}H_{38}O_4$	371	1.05108	< 0.001
33	Sucrose	900	24.21	57-50-1	$C_{12}H_{22}O_{11}$	361	1.02292	< 0.001
34	Cellobiose	870	24.94	528-50-7	$C_{12}H_{22}O_{11}$	160	1.01861	< 0.001
35	Trehalose	849	25.05	99-20-7	$C_{12}H_{22}O_{11}$	67	1.07716	0.045
36	Gentiobiose	917	25.53	554-91-6	$C_{12}H_{22}O_{11}$	204	1.07646	< 0.001
37	Melibiose	920	25.90	66009-10-7	$C_{12}H_{24}O_{12}$	204	1.07686	< 0.001
38	Raffinose	881	29.83	512-69-6	$C_{18}H_{32}O_{16}$	204	1.05837	< 0.001
39	1-Kestose	850	29.92	470-69-9	$C_{19}H_{34}O_{16}$	230	1.02229	< 0.001

[a] Numbered as in the retention time.

Supplementary Table S4. Core microbes based on Pearson's correlation coefficient. Only the microorganisms negative correlation with *Acinetobacter*, *Klebsiella*, and *Enterococcus* are shown in the table.

Genus 1	Genus 2	Correlation coefficients (Cor)	*P* < value
Weissella	*Acinetobacter*	-0.73142	0.025124
Weissella	*Klebsiella*	-0.79802	0.009945
Weissella	*Enterococcus*	-0.91246	0.000599

Cor < -0.7, $P < 0.05$
